# Host Niches and Defensive Extended Phenotypes Structure Parasitoid Wasp Communities

**DOI:** 10.1371/journal.pbio.1000179

**Published:** 2009-08-25

**Authors:** Richard Bailey, Karsten Schönrogge, James M. Cook, George Melika, György Csóka, Csaba Thuróczy, Graham N. Stone

**Affiliations:** 1 Institute of Evolutionary Biology, School of Biology, University of Edinburgh, Edinburgh, United Kingdom; 2 Department of Animal Ecology, Evolutionary Biology Centre, Uppsala University, Uppsala, Sweden; 3 Centre for Ecology and Hydrology, CEH Wallingford, Wallingford, United Kingdom; 4 Division of Biology, Imperial College London, Ascot, United Kingdom; 5 Centre for Population Biology, Imperial College London, Silwood Park Campus, Ascot, United Kingdom; 6 School of Biological Sciences, Whiteknights, University of Reading, Reading, United Kingdom; 7 Systematic Parasitoid Laboratory, Vas County Plant Protection and Soil 15 Conservation Service, Köszeg, Hungary; 8 Hungarian Forest Research Institute, Mátrafüred Research Station, Mátrafüred, Hungary; 9 Malomarok, Köszeg, Hungary; Cornell University, United States of America

## Abstract

Oak galls are spectacular extended phenotypes of gallwasp genes in host oak tissues and have evolved complex morphologies that serve, in part, to exclude parasitoid natural enemies.

Parasitoids and their insect herbivore hosts have coevolved to produce diverse communities comprising about a third of all animal species. The factors structuring these communities, however, remain poorly understood. An emerging theme in community ecology is the need to consider the effects of host traits, shaped by both natural selection and phylogenetic history, on associated communities of natural enemies. Here we examine the impact of host traits and phylogenetic relatedness on 48 ecologically closed and species-rich communities of parasitoids attacking gall-inducing wasps on oaks. Gallwasps induce the development of spectacular and structurally complex galls whose species- and generation-specific morphologies are the extended phenotypes of gallwasp genes. All the associated natural enemies attack their concealed hosts through gall tissues, and several structural gall traits have been shown to enhance defence against parasitoid attack. Here we explore the significance of these and other host traits in predicting variation in parasitoid community structure across gallwasp species. In particular, we test the “Enemy Hypothesis,” which predicts that galls with similar morphology will exclude similar sets of parasitoids and therefore have similar parasitoid communities. Having controlled for phylogenetic patterning in host traits and communities, we found significant correlations between parasitoid community structure and several gall structural traits (toughness, hairiness, stickiness), supporting the Enemy Hypothesis. Parasitoid community structure was also consistently predicted by components of the hosts' spatiotemporal niche, particularly host oak taxonomy and gall location (e.g., leaf versus bud versus seed). The combined explanatory power of structural and spatiotemporal traits on community structure can be high, reaching 62% in one analysis. The observed patterns derive mainly from partial niche specialisation of highly generalist parasitoids with broad host ranges (>20 hosts), rather than strict separation of enemies with narrower host ranges, and so may contribute to maintenance of the richness of generalist parasitoids in gallwasp communities. Though evolutionary escape from parasitoids might most effectively be achieved via changes in host oak taxon, extreme conservatism in this trait for gallwasps suggests that selection is more likely to have acted on gall morphology and location. Any escape from parasitoids associated with evolutionary shifts in these traits has probably only been transient, however, due to subsequent recruitment of parasitoid species already attacking other host galls with similar trait combinations.

## Author Summary

Herbivorous insects, such as the wasps that induce trees to make galls, and the parasitoids that attack (and ultimately kill) the wasps comprise about a third of all animal species, but it remains unclear what determines the structure of these complex coevolving communities. Here, we analyzed 48 parasitoid communities attacking different cynipid wasps that live and feed on oak trees. These communities are diverse and “closed,” with each centered upon the characteristic gall induced by a given cynipid wasp species. The often spectacular and complex galls are extended phenotypes of gallwasp genes and have been suggested to evolve as gallwasp defenses against their parasitoid enemies—“the Enemy Hypothesis.” Our analysis showed that similar parasitoid communities occurred on galls with similar structural traits (e.g., toughness, hairiness, stickiness), supporting the Enemy Hypothesis. We also found similar communities on galls that co-occur frequently in time and space; in particular, those occurring on the same oak species and same plant organ (e.g., leaf, bud, seed). Our results suggest that cynipid wasps might escape particular parasitoids via evolutionary shifts in the structure or location of their galls. However, escape may often be transient due to recruitment of new enemies already attacking other host galls with similar trait combinations.

## Introduction

Identifying the processes that structure communities remains one of the fundamental challenges facing ecology [1]–[5] and greatly influences our ability to predict the effects of species invasions and extinctions [6]–[8]. However, the issues are complex, and recent reviews have emphasised the need for new approaches to understanding the ecology and evolution of communities [9]–[11]. Two important emerging themes are (i) the effects of adaptive trait variation at one trophic level upon other levels [1],[2],[5],[12],[13] and (ii) the roles of evolutionary history and phylogenetically conserved traits in determining current community structure [4],[11],[14],[16].

Here we address these issues in a study that focuses on diverse, clearly defined communities of parasitoid wasps attacking insect herbivore hosts. Understanding the processes structuring host–parasitoid communities is important because parasitoid wasps and their insect hosts comprise about one-third of all animal species, and more than 50% of all terrestrial animal species [17]. Parasitoids also play a major role in regulating populations of their insect hosts, and the ecosystem service they provide in reducing losses to herbivores and stored product pests is estimated at billions of dollars annually [1],[2],[8],[15],[16]. Detailed studies of single insect host species have shown that variation in host traits, including feeding location, feeding mode, and host plant species [1],[2],[15],[16], influences the mortality imposed by parasitoids. However, much less is known of the effects of host trait evolution on parasitoid community composition [5],[18]. Addressing this issue requires examination of patterns in parasitoid communities across host species, and because related host species may share both similar traits and parasitoid communities through shared common ancestry [19],[20], this in turn requires explicit consideration of host phylogeny.

We studied the parasitoid wasp communities associated with a major radiation of herbivorous insects—cynipid gallwasps (*Hymenoptera*, *Cynipidae*) on oak trees (*Quercus* species)—a host taxon with some 1,000 herbivore species, distributed primarily in northern temperate regions [24]. These communities are excellent test subjects because they are diverse and well studied [21]–[29], and the vast majority of the associated parasitoids attack only oak gallwasps [21],[22],[24],[30],[31]. The communities are thus ecologically “closed” and may meaningfully be considered in isolation.

**Figure 1:**
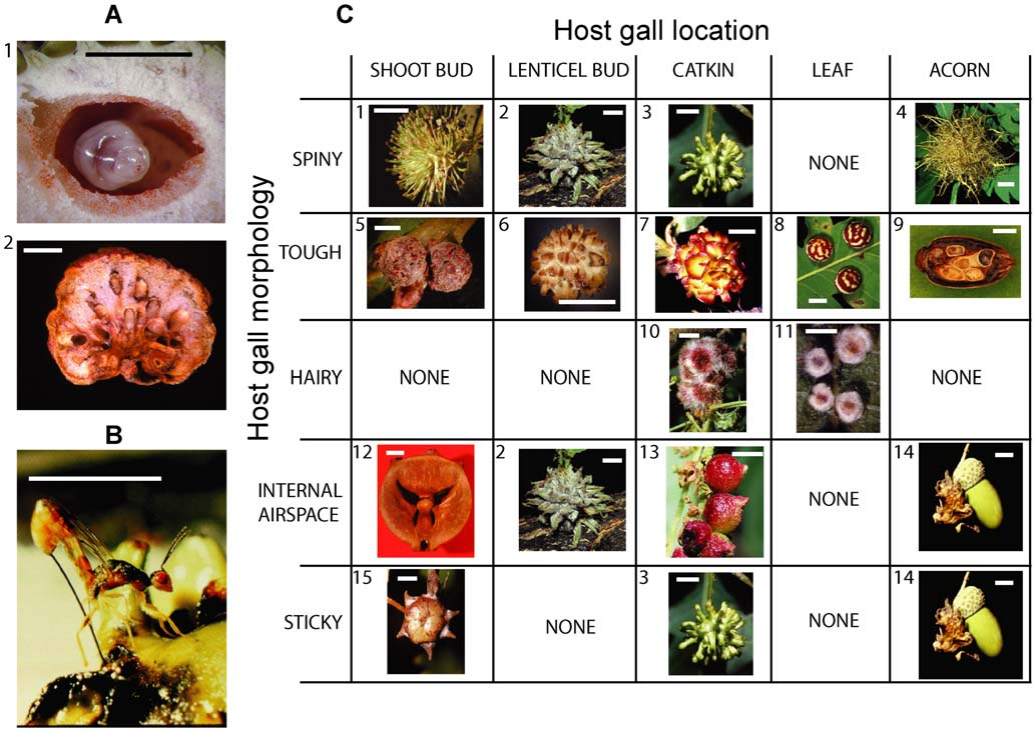
Resource availability and trait variation in oak cynipid galls. (A) Gallwasp larvae. 1. A gallwasp larva (*Andricus lucidus* asexual generation) in its larval chamber. 2. Multiple larvae in the multilocular sexual generation gall of *Biorhiza pallida*. (B) One of the parasitoids in this study, *M. stigmatizans* (Torymidae) drilling through the wall of an oak cynipid gall. (C) Matrix showing some of the diversity in defensive gall morphologies [23] and gall locations represented by species in this study, with examples (sg, sexual generation; ag, asexual generation): 1. *A. lucidus* (ag). 2. *A. hartigi* (ag). 3. *A. grossulariae* (ag). 4. *A. caputmedusae* (ag). 5. *A. lignicolus* (ag). 6. *A. gemmeus* (ag). 7. *A. lucidus* (sg). 8. *Cynips longiventris* (ag). 9. *Callirhytis glandium* (ag). 10. *Dryocosmus nitidus* (sg). 11. *Neuroterus lanuginosus*. 12. *A. quercustozae* (ag). 13. *A. grossulariae* (sg). 14. *A. quercuscalicis* (ag). 15. *A. coronatus* (ag). Scale bar = 5 mm in all images.

Oak gallwasps induce the development of spectacular galls (Figures 1 and S2), which though comprising plant tissues represent the extended phenotypes of gallwasp genes [23],[32]. Parasitoids inflict high mortality on their gallwasp hosts [24],[27],[28],[33], and selection should favour adaptive host traits that reduce parasitism [27],[34]. Since all parasitoid attack involves oviposition through gall tissues, gallwasp genes that induce gall structures that reduce parasitoid attack rates should be favoured by selection—a view encapsulated in the Enemy Hypothesis [1],[2],[27],[33]. This hypothesis is supported by studies of the impact of variation in gall morphology within galler species, and is also compatible with demonstrated convergent evolution of several of the same traits (Table 1) in gallwasps [23]–[26]. These defensive extended phenotypes drive reciprocal phenotype evolution [34],[35] in parasitoid traits, such as ovipositor length, which may limit access to concealed gallwasp hosts [31]. While previous studies have examined the role of other host defences (such as warning coloration and cuticular coatings of hair or spines (e.g., [5]) and grooming behaviours [36]), we here examine the impact of diversity in extended phenotypes in predicting variation in parasitoid community structure among a closely related group of herbivores.

**Table 1:** Summary of host characters used as explanatory variables in analyses of parasitoid community composition.

Variable	Category	Type	Character Variation
Gall hairiness	Morphology	Binary	Galls either have a smooth or hairy (defensive) surface. Labile.
Gall spininess	Morphology	Binary	Galls are either spineless or covered with spines (defensive). Labile.
Gall toughness	Morphology	Categorical	Four levels, increasing in toughness from 1 to 4. High toughness is defensive. Labile.
Gall stickiness	Morphology	Binary	Galls are either coated with sticky resin (defensive) or not. Labile.
Gall internal airspace	Morphology	Binary	Galls are either solid or have an internal airspace surrounding the larval chamber (defensive). Labile.
Host gall size	Morphology	Continuous	The volume of each mature gall type, with gall inducer larval chamber subtracted.
Host resource size	Resource availability	Continuous	The volume of each fully developed host gallwasp larva.
Gall locularity	Resource availability	Binary	Galls either contain a single host gallwasp larva, or >1. Labile.
Mean number of hosts/gall	Resource availability	Continuous	Mean number of parasitoids emerging from galls producing at least one parasitoid.
Organ galled	Spatiotemporal niche	Categorical	Gall location on the oak host, either shoot bud, dormant bud on the trunk (lenticel), acorn, leaf, catkin, or shoot. Labile.
Oak section	Spatiotemporal niche	Binary	Galls develop either on oaks in the section Cerris (*Q. cerris*) or in the section Quercus (*Q. petraea*, *Q. pubescens*, and *Q. robur*)^a^. Labile.
Season of development	Spatiotemporal niche	Continuous	The week, starting at April 1st, that the gall was first observed to start development.
Persistence	Spatiotemporal niche	Continuous	The mean duration of gall development, in weeks.
Sample size	Sampling effort	Continuous	Total number of parasitoids emerging from galls of a given type.

These are categorised as describing gall morphology, host resource availability, or gall spatiotemporal niche. Character states for all gall types are given in Table S2. Gall morphology character states were defined as defensive on the basis of their demonstrated efficacy in single species studies [27],[34]. Categorical characters with many state changes in our species set [21],[23]–[25],[41],[43] provide multiple independent contrasts and are labelled as labile.

a Please see Material and Methods for justification of classifying oak hosts at the section rather than species level.

Because the parasitoid communities attacking oak gallwasps comprise both specialists (those attacking a small subset of available host gall types) and generalists (those attacking many host gall types) [21],[22],[24],[28],[30],[31], we can also ask which of these groups drive any host-associated community structure. This is important because whereas changes in host traits influencing specialist enemies are likely to influence only a small number of species in these foodwebs, those influencing vulnerability to attack by generalists may have both major direct and indirect (apparent competition [9]) influences on many species in the web. Further, Askew [22] predicted that the richness of oak gallwasp communities would be maintained by partitioning of generalist parasitoids among different gall phenotypes.

We emphasise two key phases in successful parasitoid attack—host detection and host exploitation [1],[2],[15],[16],[34]. For parasitoids of herbivorous insects, host detection requires searching the right part of the right plant at the right time, while exploitation involves overcoming any host defences and the ability to develop on the host resources available [15],[16],[34]. We can, in turn, divide host traits into three major groups (Table 1), each of which has been invoked repeatedly [1],[2],[5],[15],[16],[19],[21],[22],[27],[30],[34] as a key determinant of parasitoid community structure: (i) Spatiotemporal niche traits describe the distribution of hosts in space (oak taxon galled, location of the gall on the oak) and time (season and duration of development), and determine the likelihood of detection by parasitoids. (ii) Resource traits represent the quality of the host resource per gall (host size, number of hosts per gall) potentially available to parasitoids. (iii) Gall morphology traits capture variation in the structure of gall tissues parasitoids must penetrate to access host resources, potentially acting as direct defences against particular natural enemies (the Enemy Hypothesis [27],[33]). These three groups of traits influence parasitoid success in host detection (spatiotemporal niche) and host exploitation (resource, morphology), respectively.

Here we compare the parasitoid communities induced by 40 gallwasp species (Table S1) at five replicate sites across Hungary (Figure S1), a known ancient centre of oak cynipid diversity [37],[38]. Oak gallwasp lifecycles involve obligate alternation between a spring sexual generation and a summer asexual generation [24],[39], each of which induces a gall with a characteristic morphology that develops on a characteristic plant organ (e.g., bud, leaf, flower, fruit, root) of a specific oak taxon [23]–[26]. Exemplar gall phenotypes are shown in Figure 1, and gall morphologies and character states for all species and generations are shown in Figure S2 and Table S2. Sexual and asexual gallwasp generations have long been known to support different parasitoid communities [21],[22],[28],[40], a feature of gallwasp ecology that here we establish quantitatively in ancient refuge communities for the first time. Because the two generations of the gallwasp lifecycle also show independent evolution of morphological traits, gall locations and host oak associations [23],[25],[26],[41], we examine patterns in associated parasitoid communities in each generation separately. Specifically, we ask whether similar parasitoid communities evolve on hosts with similar gall morphology traits (as predicted by the Enemy Hypothesis), on hosts occupying similar spatiotemporal niches, or on hosts providing similar levels of resource per gall. Further, we ask whether any host-associated community structure is driven by the preferences of generalist natural enemies, as predicted by Askew [22].

**Figure 2:**
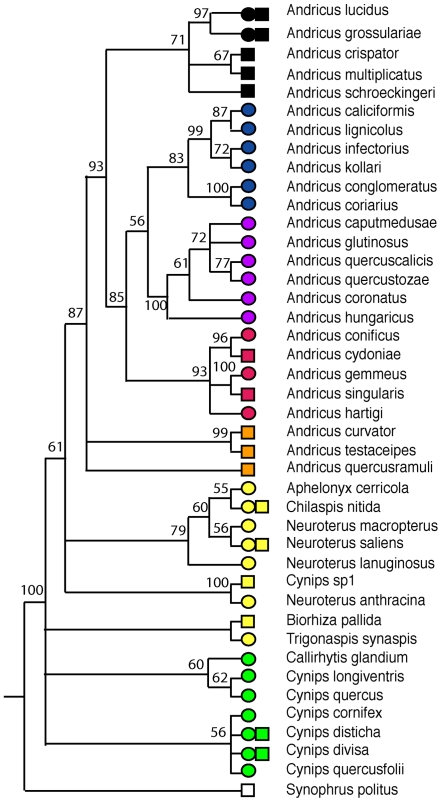
Phylogenetic relationships between host gallwasps. The mitochondrial DNA sequence phylogeny of host gallwasps, presented as a cladogram with node support shown by posterior probabilities in Bayesian analyses (see Materials and Methods). Coloured symbols at branch tips indicate gallwasp clade membership (following 23,25,39), allowing recognition of phylogenetic patterns in Figure 3. The shape of the symbol indicates the generation included in our analysis (circle, asexual; square, sexual).

A key issue in analyses of patterns across species is the fact that host trait values (both gall phenotypes and associated parasitoid communities) cannot be regarded as statistically independent, but are linked by phylogenetic patterns of shared common ancestry [5],[18],[19],[23],[25],[36],[41],[42]. To assess the strength of any phylogenetic patterning in variables of interest, we generated a molecular phylogeny of the host gallwasp species (see Materials and Methods), and then used matrix correlation analyses (MCA; see Materials and Methods) to test the significance of correlations between pairwise genetic distance between species, and pairwise similarity in phenotypic and community traits. We then use two parallel approaches that control for phylogenetic nonindependence to examine patterns within each generation (see Materials and Methods). First, we included host relatedness (shown visually in Figure 2) as a covariate in MCA of host traits and parasitoid communities. Second, we controlled for phylogenetic nonindependence using phylogenetically independent contrasts in phylogenetic regression analysis (PRA) [42]. We present results separately for each of our five study sites, and for all five sites pooled (see Materials and Methods). We predict that host traits with a key role in structuring parasitoid communities should have consistent significant effects across these different datasets.

## Results

### Sexual and Asexual Gallwasp Generations Support Significantly Different Communities

**Figure 3:**
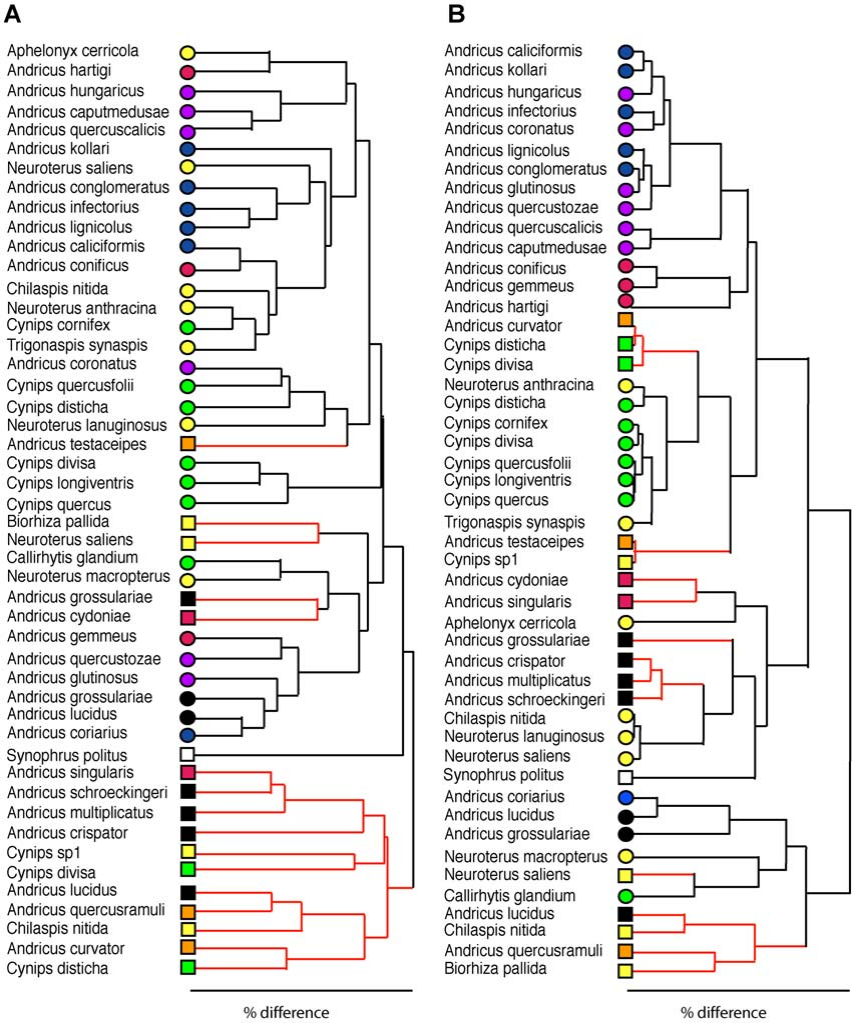
Cluster analyses showing similarity in parasitoid community composition and gall phenotypes. Cluster analyses showing similarities between gall types in (A) parasitoid assemblage composition, and (B) spatiotemporal niche character states (see Materials and Methods). Colours of symbols at branch tips match those for the clades in Figure 2. Sexual generation galls are indicated by square symbols and red branches.

We reared over 40,000 cynipid galls, resulting in >31,000 parasitoids belonging to 58 species (Table S3). Each gall type was attacked by between three and 30 parasitoid species, of varying host specificity. For the purposes of illustration (and not for data analysis), we divide parasitoid species among the following categories. There were nine extreme specialist parasitoid species (recorded from only one host gall type), 23 specialists (two to 11 hosts), 16 generalists (11–21 hosts), and nine extreme generalists (>20 hosts) (Table S3). Our placement of parasitoid species into these categories closely matches previous work on Western Palaearctic oak gall communities [21],[22],[24],[43]. To allow tests of correlations between host phenotypic traits and parasitoid community composition, we calculated the pairwise similarity in parasitoid community composition for all gall type pairs using Bray-Curtis scores. This common measure of similarity takes account of both the presence and the relative abundance of parasitoid species (see Materials and Methods), and for our sampled communities ranged from 0% (no parasitoid species in common) to 80% (great overlap of species). A striking feature of our data is the fact that though some parasitoids attack both asexual and sexual generation galls, communities associated with the same generation of different host species are significantly more similar than those associated with different generations of the same host species (shown visually in Figure 3; ANOSIM of Bray-Curtis scores by generation significant for all sites: *p*<0.001 at Gödöllõ, Mátrafüred, and Sopron; *p*<0.02 at Varpalota; *p*<0.05 at Szentkut). This replicates Askew's findings for younger and much less diverse postglacial oak gallwasp communities in the UK [21], and is consistent with a fundamental role for season of development in determining parasitoid community composition (see also [16]).

### Phenotypic Traits and Parasitoid Communities Are Strongly Correlated with Host Phylogeny

Parasitoid community composition and all three aspects of host phenotypes (spatiotemporal niche, host resource availability, gall morphology) are correlated with host phylogeny (Figures 2 and 3; Table 2). More closely related gallwasp hosts harboured more similar parasitoid communities in both sexual (*p*<0.05, pooled sites) and asexual generations (*p*<0.001, and significant at *p*<0.05 in four of five individual sites). Significant correlations were also always positive for spatiotemporal traits (plant organ galled, oak taxon, and gall persistence) and host resource availability (host size, Table 2). In contrast, signs of significant correlations varied among morphological traits; they were positive for toughness and gall size but negative for spininess, stickiness, and presence of an internal airspace. The negative correlations for the latter traits are consistent with previous analyses demonstrating their convergent evolution in gallwasps [23],[26]. A greater number of significant correlations was found in the asexual generation galls (Table 2), in part reflecting the greater trait diversity present in this generation (Figure S2; Table S2). These results underline the need to control for phylogenetic nonindependence in testing correlations between host traits and parasitoid community composition, even in closely related hosts.

**Table 2:** Phylogenetic patterns in host gall traits and parasitoid communities for sexual and asexual gallwasp generations.

Gall trait	Asexual	Sexual
***Gall morphology***		
**Hairiness**	—	—
**Toughness**	*p*<0.05 (+)	—
**Spininess**	*p*<0.01 (−)	—
**Stickiness**	*p*<0.05 (−)	—
**Internal airspace**	*p*<0.01 (−)	—
**Gall size**	*p*<0.01 (+)	—
***Resource availability***		
**Host resource size**	—	*p*<0.001 (+)
**Hosts/gall**	—	—
**Locularity**	—	—
***Spatiotemporal niche***		
**Plant organ galled**	*p*<0.001 (+)	—
**Oak section**	*p*<0.001 (+)	—
**Persistence**	—	*p*<0.001 (+)
**Season**	—	—

Cell entries show the significance of matrix correlations (and their sign) between host phylogenetic relatedness and gall traits.

### Gall Defences, Spatiotemporal Niche, and Resource Availability Each Structure Parasitoid Communities

Summaries of the significant variables retained in MCA and PRA analyses are shown in Tables 3 and 4, respectively. While MCAs addressed overall similarity in community composition using untransformed Bray Curtis similarities, in PRAs we used multidimensional scaling (MDS) to identify three statistically independent axes of community variation (see Materials and Methods). The results of the two analytical approaches are highly congruent and show parasitoid community composition to be influenced by host traits associated with each of gall morphology, spatiotemporal niche, and host resource (Tables 3 and 4). Explanatory power of these traits in combination can be high: in PRA, these groups of explanatory variables explained up to 62% of the deviance in a given MDS axis (Table 4). For some site and generation combinations, however, available host species allowed very few independent contrasts, limiting the degrees of freedom available for detection of patterns in the data (see legend, Table 4).

**Table 3:** Significant matrix correlations between gall traits (rows) and Bray-Curtis similarity in parasitoid assemblage composition.

Variable	Dataset	
	Asexual	Sexual
**Sample size**	1	—
**Relatedness (alone)**	***4	*
**Relatedness in MAM**	1	—
**Gall morphology**		
**Hairiness**	*2	—
**Toughness**	—	—
**Spininess**	*1	N/A
**Stickiness**	**	
**±Internal airspace**	—	N/A
**Gall size**	*2	1
***Resource availability***		
**Host size**	1	—
**Locularity**	—	1
**Hosts/gall**	*2	*1
***Spatiotemporal niche***		
**Plant organ galled**	***2	*
**Oak section**	**3	**1
**Season**	*1	—
**Persistence**	—	***1

Asterisks indicate significance for the pooled sites dataset (*, *p*<0.05; **, *p*<0.01; ***, *p*<0.001), whereas numbers indicate the number out of five individual sites showing a significant correlation (all *p*<0.05). Results are presented for asexual and sexual generations tested separately. Results for relatedness are given when this variable alone is fitted, followed in the row below by significance in a multiple regression with all other significant variables in the MAM (see Material and Methods). Entries marked N/A lack variance in the host gall trait for that row.

**Table 4:** Parameter estimates, significance, and percent deviance explained in MAMs for PRAs of parasitoid community composition (see Materials and Methods).

Dataset	MDS Axis	Spatiotemporal Niche			Resource		Gall Morphology			Sample Size	Percent Deviance Explained
		Oak Section	Organ Galled	Persistence	Locularity	Hosts/Gall	Hairiness	Toughness	Gall Size	Total Emer	
**Pooled sites, asexual 31/14**	1		−0.71^a^								18.9
	2	−0.69									13.2
	3		0.21^a^		0.81**		−1.14*			0.26**	62.1
**Pooled sites, sexual only 17/9**	1		−0.91^a^								41.9
	2					−0.57					54.7
	3										ns
**Mátrafüred, asexual only 21/11**	1	−0.37*					0.20**				6.2
	2	−0.20				0.77				−0.20	54.7
	3	0.55								0.16	37.2
**Mátrafüred, sexual only 13/7**	1										ns
	2										ns
	3			−0.25							61.4
**Gödöllö, asexual only 23/12**	1	0.34						0.96^b^**		0.13	26.7
	2										ns
	3										ns
**Gödöllö, sexual only 11/6**	1					0.57					38.3
	2										ns
	3										ns
**Szentkút, asexual only 22/11**	1							−0.78^c^	−0.0003		60.6
	2										ns
	3	−0.32					−0.68				14.5
**Sopron, asexual only, 27/13**	1										ns
	2		−0.83^d^ 0.48^a^								53.5
	3		−0.14^a^** 0.55^e^**								6.8

Only significant results are shown, separated by site and for sexual and asexual host generations. Significance level is indicated as follows: no *, *p*<0.05; *, *p*<0.003 (threshold for significance with Bonferroni correction for analyses for individual sites and generations); and **, *p*<0.001. Total emer, total number of emerging parasitoids; Hosts/gall, mean parasitoids per gall, given by total emergence/total producing from Table S2. The figures following the name of each dataset in the first column give the number of species/number of phylogenetically independent contrasts in each analysis. No significant correlations for any dataset were obtained for the following host gall variables: resource volume, spininess, stickiness, and the presence/absence of an internal airspace. No significant correlations for any variable were obtained for sexual generation galls at Szentkút, either sexual or asexual generation galls at Várpalota (insufficient degrees of freedom were available for analysis in the sexual generation), or sexual generation galls at Sopron. These analyses have four of the five lowest numbers of species and independent contrasts, with 8/5, 4/1, 13/5, and 12/6, respectively. Superscript letters show significant character states for “organ galled” locations (a–c) and gall “toughness” (d and e).

a Leaf.

b Soft.

c Very hard.

d Acorn.

e Shoot.

#### (i) Gall morphology

We obtained significant correlations with parasitoid community composition for putatively defensive gall structure traits (Tables 3 and 4): gall hairiness (MCA and PRA), gall size (MCA and PRA), gall toughness (PRA), gall spininess (MCA), and gall stickiness (MCA). All but one of the significant correlations were obtained for parasitoid communities attacking the more structurally complex and diverse asexual generation galls (see Figure 1). Significance in two or more individual site datasets was revealed for gall hairiness (PRA: three sites), gall toughness (PRA: two sites), and gall size (MCA: two sites). Our results thus show that gall traits of demonstrated defensive value within galler species also influence parasitoid community structure among species, and so support the Enemy Hypothesis for gall structural diversity.

#### (ii) Spatiotemporal niche traits

Host oak section had the most consistently significant impact on parasitoid community composition of any single host trait, with significant correlations for both generations in MCA, and for three individual site asexual generation analyses in both MCA and PRA (Tables 3 and 4). The location of the host gall within the oak (leaf, bud, acorn, etc.) was also significant in both generations, and in two individual site asexual generation analyses in both MCA and PRA. Host gall persistence (MCA for sexual generation galls) and season of development (MCA for asexual generation galls, and for one individual site asexual generation PRA) had significant but less general impacts on community structure.

#### (iii) Host resource traits

Neither analytical approach revealed significant correlations between host resource size and community composition in either generation. In contrast, aggregation of hosts (as measured by the variables locularity and hosts/gall; Tables 3 and 4) influenced community structure in both generations.

### Host Gall Traits Influence the Relative Impacts of Generalist Parasitoids

**Figure 4:**
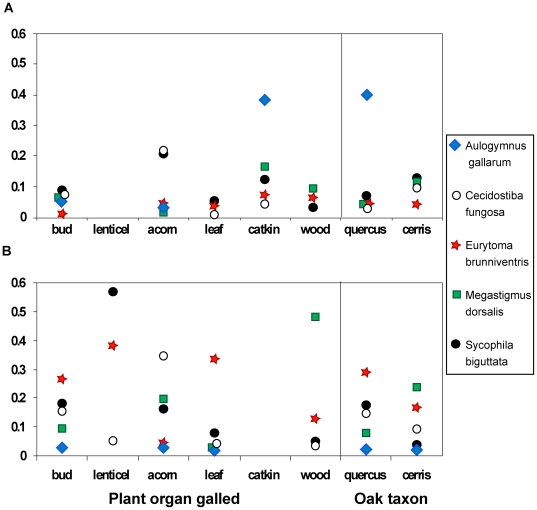
Host trait-associated variation in parasitism by generalist parasitoids in communities associated with sexual and asexual generation oak gallwasp communities. The dominance plot shows, for the five most generalist parasitoid species, the proportion individuals of a given species comprise of all emerged parasitoids ( = dominance) averaged across host gall types with specific gall locations and oak associations. Gall locations refer to the plant organ galled (the location category “wood” refers to galls integral to the main axis of shoots), while oak taxon associations refer to gall induction on species in either *Quercus* section Cerris or section Quercus sensu stricto. The selected parasitoid taxa are *A. gallarum* (Eulophidae), *C. fungosa* (Pteromalidae), *E. brunniventris* (Eurytomidae), *M. dorsalis* (Torymidae), and *S. biguttata* (Eurytomidae). The data from different host gall types have been pooled at two biologically relevant spatial scales, namely galls on different plant organs (left) and on different oak host taxa (right).

Askew [22] proposed that structuring of rich cynipid-centred communities would be mediated by variation in the ability of generalist parasitoids to exploit different host phenotypes. Our data show that the relative dominance (see Materials and Methods) of the five most generalist species attacking each gallwasp generation varies with host traits (Figure 4). For example, in the sexual generation gall communities, the parasitoid *Aulogymnus gallarum* (Eulophidae; 23 recorded host gall types) was a dominant parasitoid of catkin galls (38.5% of all parasitoid emergence), but was rare (<6%) or absent in galls developing in any other location. Similarly, it was a dominant sexual generation parasitoid of hosts on section *Quercus* oaks (40.1%), but was much rarer in hosts on section Cerris. Reversals in the relative importance of parasitoid species pairs across sexual gall locations can also be seen for *Megastigmus dorsalis* (Torymidae) and *Sycophila biguttata* (Eurytomidae). A similar and more pronounced pattern is seen in the asexual generation galls: each of the five gall locations represented in our sampling was dominated by a different generalist parasitoid (Figure 4), and the relative ranks of these parasitoids differed across hosts in the oak sections Cerris and *Quercus*. Where sampled gall types allow similar dominance analyses to be made in individual sites, the observed patterns are concordant with those across the pooled data. These results are consistent with Askew's hypothesis.

## Discussion

Previous multispecies studies of communities centred on herbivorous insects have shown that a range of host traits structure parasitoid assemblages, including food plant taxon [18],[20],[44]–[50], host feeding niche (e.g., exposed versus leaf mining versus gall inducing; [18],[48],[50]–[54]), season of development [50],[55],[56], duration of gall development [55], and intrinsic (i.e., direct behavioural and morphological) defences against parasitoid attack [5],[57],[58]. The relationships we find between host spatiotemporal niche traits and parasitoid community composition further strengthen several of these patterns. The strong separation between communities attacking spring (sexual) and summer (asexual) gallwasp generations parallels similar phenology-associated community structure in other concealed hosts, including gall midges [55] and leaf miners [50], as does the impact of host plant taxon [18],[48],[50]. The significance of gall location independent of host plant taxon parallels similar findings in communities attacking galling sawflies on willows [18]. Our findings that host aggregation (multilocularity, hosts per gall) influences community structure while host resource size does not also parallels findings in other systems [5]. The generality of these patterns implies that phenological matching commonly dictates the pool of available parasitoid species, whereas the location of hosts within specific plant taxa dictates which subset of this pool has appropriate searching behaviours to detect them [1],[2],[15],[16],[44]–[47]. Our findings suggest that species interactions, such as resource competition and multiparasitism among parasitoid species [15],[16], as well as host–parasitoid coevolution [15],[16],[59],[60], will commonly involve hosts on the same oak taxon and plant organ.

### Significant Impacts of Gall Extended Phenotypes Support the Enemy Hypothesis

One initially counterintuitive finding of studies comparing parasitoid communities associated with different host feeding niches was that despite being apparently well defended by gall tissues, gall inducers usually support richer communities and suffer higher mortality than externally feeding herbivores ([48],[51]–[53]; but see [54]). This may be because, as proposed by Stireman and Singer [5] for tachinid fly parasitoid communities, well-defended hosts suffer lower mortality from attack by generalist vertebrate (and possibly invertebrate) predators, and so represent enemy-free space [61] for their specialist parasitoids. Whether the same applies to cynipid galls and their associated parasitoids or not, the Enemy Hypothesis predicts that the high host mortality imposed by parasitoids on most insect gall inducers should drive the evolution of gall phenotypes that reduce attack by, or exclude, at least a subset of them [1],[2],[27],[33],[52],[62]. Though there is evidence that herbivore extended phenotypes do structure parasitoid assemblages across host species [18],[63], no previous studies have demonstrated an impact of the complex gall morphology traits that predict vulnerability to attack within gall-inducer species [27],[52],[53]. This led to the hypothesis that observed phenotypic diversity in some galler lineages represents the “ghost of parasitism past” [51], whose efficacy in influencing parasitoid attack has been nullified by the evolution of effective parasitoid countermeasures. Our results show that in the rich, sympatric communities of cynipid gallwasps on oaks, the gall-associated extended phenotypes of gallwasp genes can structure parasitoid communities, and so provide support for the Enemy Hypothesis. Further, they show that traits expressed in two distinct stages of the gallwasp lifecycle can influence community structure, because while gall morphology is controlled by genes expressed in the gallwasp larva, oak taxon, gall location, and the grouping of hosts within gall structures are all determined by the oviposition behaviour of the adult female [23],[25],[41].

Although we have demonstrated that gall morphology influences parasitoid community composition, none of the gall morphologies we sampled were free of parasitoids, and so none represent true enemy-free space [61]. This suggests that any enemy-free space gained by novel gall morphologies is only transient [1],[2],[27],[35]. The ability of all but seven of the parasitoid species in this study to attack multiple gall morphologies implies that parasitoids are able to circumvent some structural gall defences through behavioural or phenological plasticity [35], and consequently the coevolution of host morphological defences with parasitoid attack mechanisms is probably diffuse [15],[35],[60]. For a gallwasp, our results suggest that the best way to escape its current parasitoid community in a given generation is to shift to a new oak taxon, or to a new location on its current oak host. Which of these routes has been exploited in the evolution of gallwasp communities will depend on the relative frequency of each kind of shift during gallwasp diversification. In contrast to other gall inducers [18], oak gallwasps shift between oak lineages extremely rarely [41], while changes in gall location and morphology are more frequent [23],[25],[26]. However, when evolutionary shifts in gall location occur, they will often be a case of “out of the frying pan and into the fire,” because of subsequent detection and exploitation of novel hosts by parasitoid species already attacking other host galls resident in the same spatiotemporal niche [1],[2].

Why then are similar impacts of gall morphology not seen in the parasitoid communities associated with species-rich radiations of hosts inducing structurally complex galls on other plants (such as *Asphondyllia* gall midges on creosote bush, *Larrea tridentata*; [52])? One possibility is that gall phenotypes in these radiations do represent the “ghost of parasitism past.” An alternative is that relationships between gall morphology and associated communities may only become apparent when phylogenetic patterns are controlled for. Changes in parasitoid assemblages during evolutionary diversification of a host lineage represent the sum of phylogenetic correlation between the assemblages attacking related hosts, and the impacts of variation in any host traits influencing parasitoid attack. If phylogenetic correlations are strong (as they are in a range of host–parasitoid systems [5],[18],[56]), significant impacts of gall morphology traits may only be revealed when host phylogeny is controlled for, as we have done here. Though patterns of evolution have been examined in *Asphondyllia* gall traits [64], to our knowledge phylogenetically controlled analyses of associated parasitoid assemblages have yet to be made.

In addition to their parasitoid natural enemies, some cynipid gallwasps are also attacked by opportunist vertebrate natural enemies, including insectivorous birds [43]. Studies on other gall-inducer systems [34] have shown that birds can impose directional selection on gall traits, and although available evidence shows that parasitoids inflict the vast majority of natural enemy-imposed mortality in cynipid galls [43], it is possible that the traits we discuss here could also influence bird predation in this system.

### Gall Traits Structure Communities through Their Impact on Generalist Parasitoids

Species rich communities of insect herbivores often harbour multiple generalist parasitoids [15],[16],[44],[50],[65], raising the question of how host species richness is maintained in the face of apparent competition [9],[66]–[69]. Our results show that within each gallwasp generation, significant impacts of host traits on parasitoid community structure primarily involve generalist parasitoids (Figure 4). This argues against the existence of clearly defined tritrophic niches in gallwasp communities, in which hosts in specific niches are attacked by specific sets of natural enemies [1],[2]. However, the fact that generalist parasitoids vary in the mortality they inflict in different spatiotemporal niches makes them less generalist than their host ranges would suggest. Though we have not explicitly examined it here, one possible consequence of this is a weakening of indirect interactions (such as apparent competition) between hosts mediated by shared enemies [25],[65],[68], with potential contributions to food web stability [67],[69],[70]. Interaction networks can also be stabilised by switching of parasitoids between alternative hosts [66],[67]. If such host switching occurs in oak gall parasitoids, our results suggest that it will be primarily among hosts in the same gall generation and probably on the same oak taxon.

Further studies across guilds of natural enemies that exploit potentially coevolving hosts are needed to assess the generality of the patterns of community structure found here. Influential generalist natural enemies attacking spatiotemporally linked metacommunities of hosts may be a feature of many natural communities, as oak gall communities show many similarities with those centred on other concealed insect herbivores (such as other gall inducers, leaf miners, or stem borers) [15],[16],[22],[45]–[47],[65],[68], including many pests and potential targets for biological control. Our work underlines the need to incorporate host phylogeny into analyses of community structure, and doing so may help to predict both the natural enemies awaiting invading hosts, and the nontarget hosts of possible biocontrol agents [65].

## Materials and Methods

### Host Species and Their Gall Traits

Full names of all host gallwasp species are listed in Table S1 and gall traits are defined in Table 1. Gall morphology traits were recorded for mature galls and are listed in Table S2, and shown in Figure S2. Gall season refers to the date of the first recorded onset of development of a gall type (see Table 1). Gall persistence was measured in weeks from the onset of development until the gall inducer emerged, or the gall fell from the tree, or the end of parasitoid attack in a given year—assumed here to be the end of October on the basis of parasitoid emergence dates from our rearings.

Host abundance per gall was estimated using the mean number of parasitoids emerging from a single gall of each phenotype. Both sample size and the mean number of parasitoids per gall were ln(value +1) transformed prior to analysis.

### Parasitoid Community Data

Galls were collected between 2000 and 2003 at five field sites in Hungary (Figure S1) and reared individually in outside insectaries. At each sample site, galls were collected from >100 individual trees comprising all oak species present, separated by an average of 20–50 m, over an area of approximately 0.25 km^2^ (500 m×500 m). Each site was searched systematically and thoroughly at fortnightly intervals between April and October, and where natural host distributions allowed, each gall type was harvested as far as possible across the full site area. Galls were harvested haphazardly with respect to height and aspect, to a maximum of 8 m above ground level with a long handled pruner. Galls were always harvested in their first year of development, typically across a number of dates but also before emergence of gall inhabitants and after adequate gall growth to allow inhabitants to develop to adulthood. The emerging wasps were identified to species level and all host and parasitoid species are listed in Tables S1 and S3. The target sample size per gall type per site was 150, based on our previous work on cynipid communities [24],[28],[29]. Because of unavoidable variation in what was actually reared (Table S4), sample size was fitted as a covariate in all analyses. Pairwise similarities between parasitoid communities for use in both MCA and Analyses Of SIMilarity (ANOSIM; [71]) were calculated as Bray-Curtis similarities in PRIMER 5 (Primer-E Ltd) from standardised untransformed parasitoid abundances. We did this for individual sites and for the pooled sites dataset (i) for pooled gallwasp generations to allow us to test differences between sexual and asexual generation communities (ANOSIM), and (ii) for sexual and asexual generations separately to allow analyses of patterns within each generation (MCA). The ANOSIM analyses were carried out as a one-way design using generation as explanatory factor. The number of possible permutations was capped at 999. To allow analysis of community composition using phylogenetically independent contrasts, the Bray-Curtis matrix was decomposed into three mutually independent variables using MDS [72]. Three MDS dimensions were used for each dataset, which reduced STRESS [72] (a measure of goodness of fit) to below an acceptable threshold [72] of 0.15 in every case. Each MDS dimension was tested as a separate response variable. The parasitoid community response variables and other descriptors (MDS axes, species richness, sampling effort) are listed in Table S4. The oaks attacked by gallwasp hosts were identified to oak section, either section Cerris (*Q. cerris*) or section *Q. sensu stricto* (*Q. petraea*, *Q. pubescens*, *Q. robur*). We did not attempt to separate species within the section *Quercus* because extensive hybridisation makes definitive allocation of individuals to species using either morphological or molecular markers impossible [73]–[78]. In this we match observed patterns in oak gallwasp host specificity [25],[39],[41] and follow previous analyses of insect biodiversity on Western Palaearctic oaks [79],[80].

### Gallwasp Phylogeny

The gallwasp phylogeny was estimated from partial sequences (433 base pairs, accession numbers in Table S1) of the mitochondrial cytochrome b locus. Generation of the sequence data and selection of appropriate models of sequence evolution for this locus are discussed in detail elsewhere [39],[41]. Our phylogenetic hypothesis (working phylogeny *sensu* Grafen [42]) was generated using a Bayesian approach in MrBayes 3.0 [81] using the general time-reversible (GTR) model of sequence evolution. Trees were sampled over 10^6^ generations with an empirically determined burn-in period of 10^5^ generations before tree sampling began. Convergence of parameter estimates over each run was confirmed using Tracer [82]. As in previous phylogenetic analyses of Western Palaearctic oak gallwasps [23],[25],[41], we used the rose gallwasp *Diplolepis rosae* as the outgroup. The topology of the phylogeny used here matches very closely the results of more extensive published analyses that use a combination of mitochondrial and nuclear markers [25],[41]. GenBank (http://www.ncbi.nlm.nih.gov/Genbank) accession numbers for all sequences used in our analysis are listed in Table S1.

### Analysis of Phylogenetically Independent Contrasts

We used PRA [42] for multiple regression of phylogenetically independent contrasts in GLIM 4.0. This approach uses a user-defined working phylogeny to structure a generalised linear modelling analysis in which significance of each explanatory variable was tested in turn while controlling for all others. The approach assumes a normal distribution during model fitting, and where necessary, variables were transformed to meet this assumption (see “Host Species and Their Gall Traits” above). To minimise the impact of phylogenetic uncertainty on the regression procedure [42], we took the conservative approach of collapsing nodes with a posterior probability of <70% into polytomies. Grafen's default “figure 2 method” [42] was used to determine the initial distribution of branch lengths (node height = (*i* − 1)/(*n* − 1), where *n* = number of species and *i* = number of species below that node in the phylogeny). Minimal adequate models (MAM) were determined by stepwise removal of all nonsignificant (*p*>0.05) variables. Where the multilevel factors “plant organ galled” and “gall toughness” were retained in models, the categories were split into a series of binary variables. These were then tested in all possible combinations, controlling for other significant variables, to reveal significant categories. In analyses for separate generations and sites, significance levels were adjusted for multiple tests using the Bonferroni correction (corrected threshold *p* = 1 − (1 − alpha)^1/k^ where *k* is the number of tests and alpha is the desired threshold value of 5%). The numbers of species and independent contrasts in each analysis are given in Table 4.

### MCA

In these analyses, host relatedness was incorporated as a covariate and estimated as (1 – the GTR model proportional sequence divergence between host species pairs) for the cytochrome *b* data, calculated using PAUP* [83]. Pairwise divergences between species for this gene closely parallel those in a nuclear gene (long wavelength opsin; [41]), suggesting that this is an appropriate measure of phylogenetic relatedness. MCA was carried out with simple or partial Mantel permutation tests in FSTAT [84], using 2,000 permutations and following Manly [85]. As in the PRA, nonsignificant variables were removed from each full model to leave the MAM. Analyses were carried out for pooled and single sites, for separate generations. Similarities in gall traits were calculated using the Manhattan method for continuous variables and the Jaccard index for binary variables [86].

### Analyses of Parasitoid Dominance

We used a SIMPER analysis in PRIMER5 to reveal which species of parasitoid accounted for the majority of pairwise Bray-Curtis similarity in associated parasitoid communities of host gall types. Most of the variation could be attributed to five species: *A. gallarum* (Eulophidae), *Cecidostiba fungosa* (Pteromalidae), *Eurytoma brunniventris* (Eurytomidae), *M. dorsalis* (Torymidae), and *S. biguttata* (Eurytomidae). All five species are extreme generalists and were recorded from more than 20 host gall types in this study (Table S3). To visualise variation in the impact of these species across galls with different traits, we (i) calculated the dominance of each species in each host gall type as the proportion of individuals of that species of the total of emerging parasitoids, and then (ii) averaged the dominance values for each parasitoid species across host gall types sharing each selected trait of interest.

## Supporting Information

Figure S1 **Field sampling sites.** The five sites sampled (latitude and longitude in decimal degrees) were Mátrafüred (47.83 N, 19.97 E), Gödöllõ (47.6 N, 19.35 E), Szentkút (47.98 N, 19.8 E), Várpalota (47.20 N, 18.13 E), and Sopron (47.68 N, 16.57 E).
(0.12 MB TIF)

Figure S2 **The host gall phenotypes in this study.** The following images show the mature phenotypes of all 48 gall types in our study, numbered according to the list above. In each image the scale bar is 1 cm long.
(17.64 MB PDF)

Table S1 **Full names and GenBank accession numbers of the host gallwasps.** The number by each species and gall generation (A, asexual; S, sexual) identifies it in Figure S2 and Tables S2 and S4.
(0.03 MB DOC)

Table S2 **Gall scores for explanatory variables.** Key to columns: Gall vol., Gall cortex volume (mm^3^); Hair, hairiness; Hard, toughness (1, soft; 2, semi-soft; 3, hard; 4, very hard); Loc, locularity (M, multilocular; U, unilocular); N, absent; Oak, Oak section (Q, *Quercus* section Quercus; C, *Quercus* section Cerris); Org, host organ galled (B, bud; C, catkin; A, acorn; L, lenticel bud; Lf, leaf; S, shoot); Phen, persistence; Res, resource volume (mm^3^); Seas, season; Space, presence/absence of an internal airspace; Spine, spininess; Stick, stickiness; Y, present.
(0.11 MB DOC)

Table S3 **Parasitoid species list.** The full names and family affiliations of all parasitoid species sampled are given below. All are members of the superfamily Chalcidoidea. The families represented are Eulophidae (Eul), Eupelmidae (Eup), Eurytomidae (Eury), Ormyridae (Orm), Pteromalidae (Pter), and Torymidae (Tor). A full list of the parasitoid composition of galls of each host is available from the authors by request.
(0.04 MB DOC)

Table S4 **Gall scores for response (community) and sampling variables for pooled-sites analyses in each of (i) sexual generation galls, and (ii) asexual generation galls.** Key to variables: Galls producing, total number of galls producing parasitoids; No. emerged, total number of emerging parasitoids; Richness, species richness; MDS axes 1–3 are values for three mutually independent MDS axes describing community composition.
(0.11 MB DOC)

## Abbreviations

MAM: minimal adequate model
MCA: matrix correlation analysis
MDS: multidimensional scaling
PRA: phylogenetic regression analysis
