# Supplementary figures and images for: Host Niches and Defensive Extended Phenotypes Structure Parasitoid Wasp Communities
Source: PLoS Biol. 2009 Aug 25;7(8):e1000179. doi: 10.1371/journal.pbio.1000179 (PMC2719808; doi:10.1371/journal.pbio.1000179)

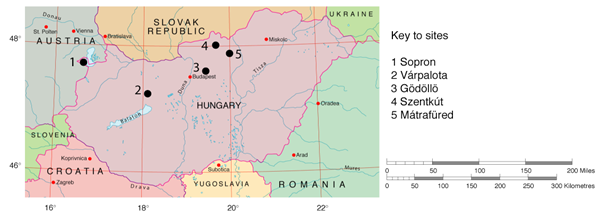

Supplement: Figure S1 — Field sampling sites. The five sites sampled (latitude and longitude in decimal degrees) were Mátrafüred (47.83 N, 19.97 E), Gödöllõ (47.6 N, 19.35 E), Szentkút (47.98 N, 19.8 E), Várpalota (47.20 N, 18.13 E), and Sopron (47.68 N, 16.57 E). (0.12 MB TIF) [file pbio.1000179.s001.tif]
