# Supplementary material for: Host Niches and Defensive Extended Phenotypes Structure Parasitoid Wasp Communities
Source: PLoS Biol. 2009 Aug 25;7(8):e1000179. doi: 10.1371/journal.pbio.1000179 (PMC2719808; doi:10.1371/journal.pbio.1000179)

Figure S2. The host gall phenotypes in this study. The following images show the mature gall phenotypes of all 48 species in our study, numbered according to Table S1. In each image the scale bar is 1cm long.

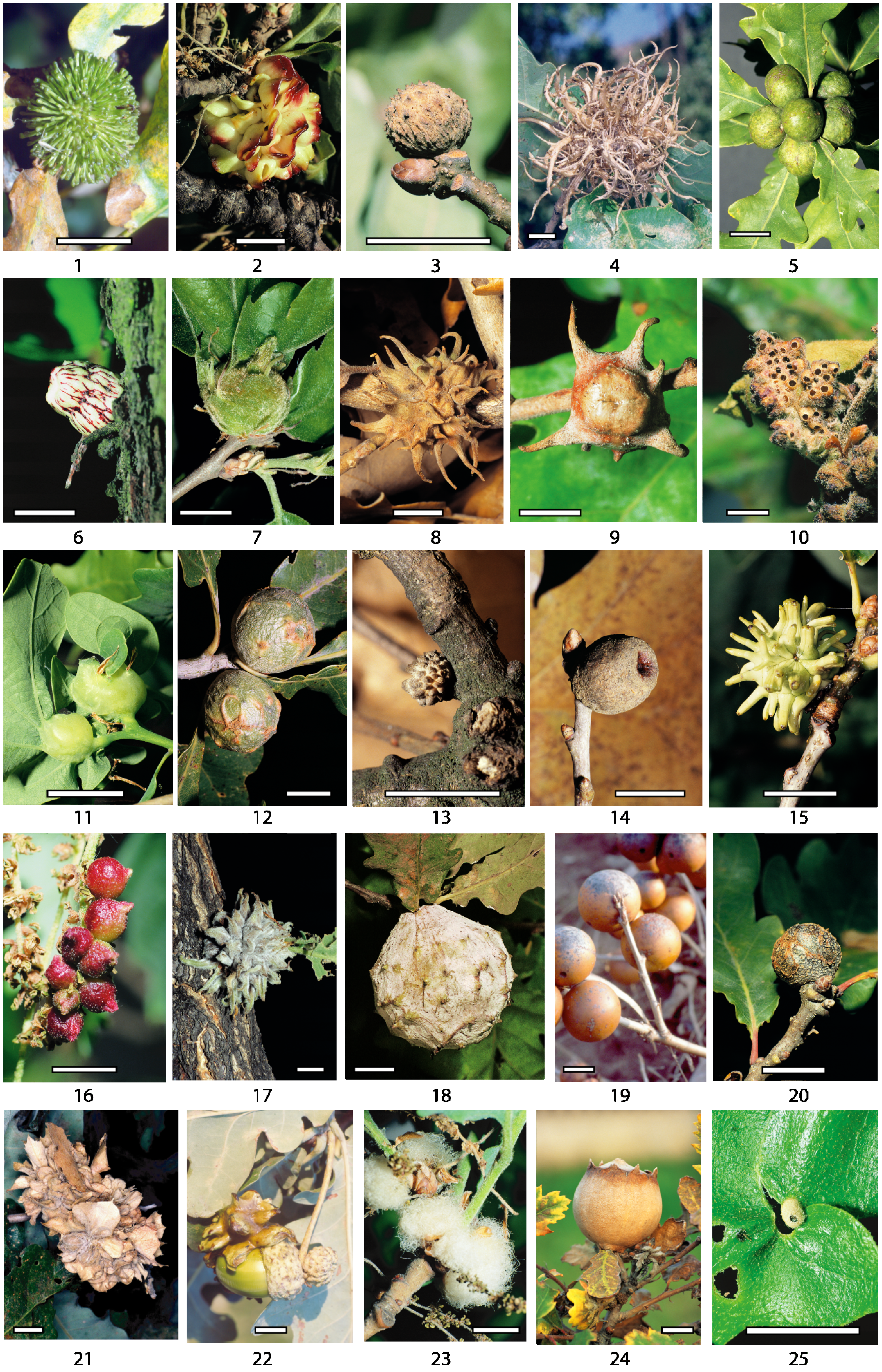

Figure S2 (cont.). The host gall phenotypes in this study. In each image the scale bar is 1cm long.

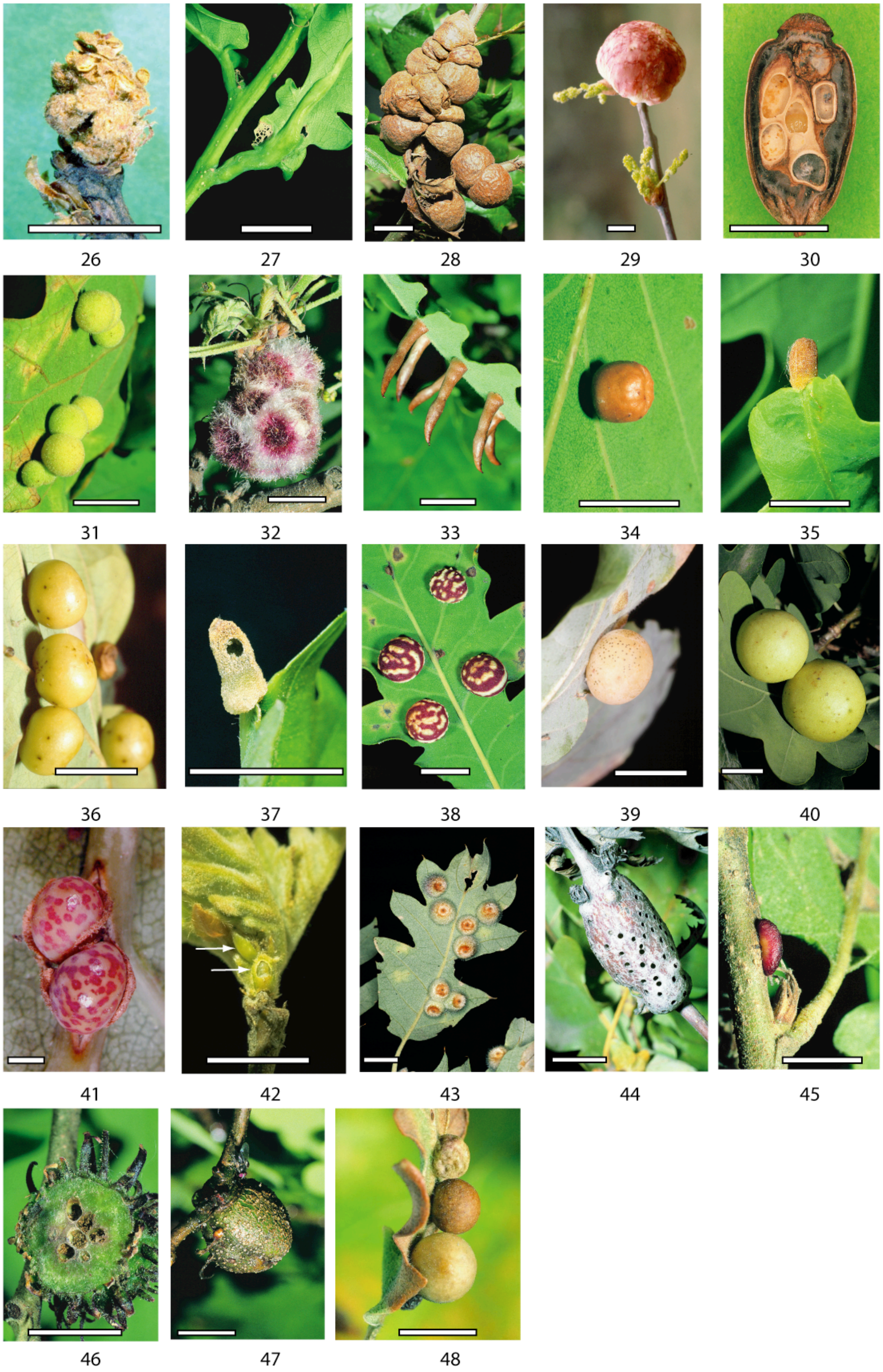

Supplement: Figure S2 — The host gall phenotypes in this study. The following images show the mature phenotypes of all 48 gall types in our study, numbered according to the list above. In each image the scale bar is 1 cm long. (17.64 MB PDF) [file pbio.1000179.s002.pdf]
