# Supplementary material for: Host Niches and Defensive Extended Phenotypes Structure Parasitoid Wasp Communities
Source: PLoS Biol. 2009 Aug 25;7(8):e1000179. doi: 10.1371/journal.pbio.1000179 (PMC2719808; doi:10.1371/journal.pbio.1000179)
Supplement: Table S1 — Full names and GenBank accession numbers of the host gallwasps. The number by each species and gall generation (A, asexual; S, sexual) identifies it in Figure S2 and Tables S2 and S4. (0.03 MB DOC) [file pbio.1000179.s003.doc]

1 *Andricus lucidus* A (Hartig, 1843), AJ228464

2 *Andricus lucidus* S (Hartig, 1843), AJ228464

3 *Andricus caliciformis* A (Giraud, 1859), AJ228455

4 *Andricus caputmedusae* A (Hartig, 1843), AJ228456

5 *Andricus conglomeratus* A (Giraud, 1859), AJ228468

6 *Andricus conificus* A (Hartig, 1843), AJ228460

7 *Andricus conificus* S (Hartig, 1843) AJ228460

(=*Andricus cydoniae* Giraud, 1859)

8 *Andricus coriarius* A (Hartig, 1843), AJ228458

9 *Andricus coronatus* A (Giraud, 1859), AJ228461

10 *Andricus crispator* S Tschek, 1871, AF539560

11 *Andricus curvator* S Hartig, 1840, AJ228453

12 *Andricus infectorius* A (Hartig, 1843), AJ228466

13 *Andricus gemmeus* A (Giraud, 1859), AJ228474

14 *Andricus glutinosus* A (Giraud, 1859), AF539563

15 *Andricus grossulariae* A Giraud, 1859, AJ228470

16 *Andricus grossulariae* S Giraud, 1859, AJ228470

17 *Andricus hartigi* A (Hartig, 1843), AJ228454

18 *Andricus hungaricus* A Hartig 1843, AJ228448

19 *Andricus kollari* A (Hartig, 1843), AJ228466

20 *Andricus lignicolus* A (Hartig, 1840), AJ228451

21 *Andricus multiplicatus* S Giraud, 1859, submitted to Genbank

22 *Andricus quercuscalicis* A (Burgsdorf 1783), AJ228459

23 *Andricus quercusramuli* S (Linnaeus, 1761), AF481706

24 *Andricus quercustozae* A (Bosc 1792), AJ228467

25 *Andricus schroeckingeri* S Wachtl, 1876, AJ131067

26 *Andricus singularis* S Mayr, 1870, submitted to Genbank

27 *Andricus testaceipes* S Hartig, 1840, submitted to Genbank

28 *Aphelonyx cerricola* A (Giraud, 1859), AJ228476

29 *Biorhiza pallida* S (Olivier, 1791), AJ228481

30 *Callirhytis glandium* A (Giraud, 1859), AF539590

31 *Chilaspis nitida* A (Giraud, 1882), AJ131069

32 *Chilaspis nitida* S (Giraud, 1882), AJ131069

33 *Cynips cornifex* A Hartig 1843, AJ228479

34 *Cynips disticha* A Hartig, 1840, AF539580

35 *Cynips disticha* S Hartig, 1840, AF539580

36 *Cynips divisa* A Hartig, 1840, AJ228477

37 *Cynips divisa* S Hartig, 1840, AJ228477

38 *Cynips longiventris* A Hartig, 1840, AF539581

39 *Cynips quercus* A (Fourcroy, 1785), AJ228478

40 *Cynips quercusfolii* A (Linnaeus, 1758), AF539586

41 *Neuroterus anthracinus* A (Curtis, 1838) submitted to Genbank

42 *Neuroterus anthracinus* S (Curtis, 1838) submitted to Genbank

43 *Neuroterus lanuginosus* A Giraud, 1859, AF539587

44 *Pseudoneuroterus macropterus*A (Hartig, 1843) AJ131070

45 *Neuroterus saliens* A (Kollar, 1857) AF539589

46 *Neuroterus saliens* S (Kollar, 1857) AF539589

47 *Synophrus politus* S Hartig, 1843 submitted to Genbank

48 *Trigonaspis synaspis* A (Hartig, 1841), AF539591
