# Supplementary material for: Host Niches and Defensive Extended Phenotypes Structure Parasitoid Wasp Communities
Source: PLoS Biol. 2009 Aug 25;7(8):e1000179. doi: 10.1371/journal.pbio.1000179 (PMC2719808; doi:10.1371/journal.pbio.1000179)
Supplement: Table S2 — Gall scores for explanatory variables. Key to columns: Gall vol., Gall cortex volume (mm3); Hair, hairiness; Hard, toughness (1, soft; 2, semi-soft; 3, hard; 4, very hard); Loc, locularity (M, multilocular; U, unilocular); N, absent; Oak, Oak section (Q, Quercus section Quercus; C, Quercus section Cerris); Org, host organ galled (B, bud; C, catkin; A, acorn; L, lenticel bud; Lf, leaf; S, shoot); Phen, persistence; Res, resource volume (mm3); Seas, season; Space, presence/absence of an internal airspace; Spine, spininess; Stick, stickiness; Y, present. (0.11 MB DOC) [file pbio.1000179.s004.doc]

| Gall type | Oak | Org | Pers | Seas | Loc | Res | Hair | Spine | Stick | Hard | Space | Gall size |
| --- | --- | --- | --- | --- | --- | --- | --- | --- | --- | --- | --- | --- |
| 1 | Q | B | 14 | 16 | M | 0.6 | N | Y | Y | 4 | N | 14.0 |
| 2 | C | C | 8 | 6 | M | 2.3 | N | N | Y | 3 | N | 116.6 |
| 3 | Q | B | 10 | 11 | U | 10.5 | N | N | N | 4 | N | 176.9 |
| 4 | Q | A | 17 | 11 | U | 5.4 | N | Y | Y | 4 | N | 654.2 |
| 5 | Q | B | 19 | 11 | U | 10.5 | N | N | N | 2 | N | 785.8 |
| 6 | Q | L | 12 | 18 | U | 8.7 | N | N | N | 2 | N | 359.2 |
| 7 | C | B | 4 | 4 | M | 0.7 | N | N | N | 1 | N | 81.8 |
| 8 | Q | B | 16 | 14 | M | 3.6 | N | N | N | 4 | N | 81.2 |
| 9 | Q | B | 12 | 18 | U | 2.3 | N | N | Y | 3 | Y | 128.7 |
| 10 | C | Lf | 5 | 4 | U | 0.7 | N | N | N | 2 | N | 6.6 |
| 11 | Q | Lf | 2 | 4 | U | 0.7 | N | N | N | 1 | N | 19.9 |
| 12 | Q | B | 14 | 14 | U | 11.8 | N | N | N | 3 | N | 1841.5 |
| 13 | Q | L | 5 | 19 | U | 0.7 | N | N | N | 1 | N | 13.5 |
| 14 | Q | B | 20 | 10 | U | 2.3 | N | N | Y | 4 | Y | 276.0 |
| 15 | Q | A | 8 | 18 | M | 2.3 | N | N | Y | 4 | N | 116.6 |
| 16 | C | C | 4 | 4 | U | 0.7 | N | N | N | 2 | N | 43.9 |
| 17 | Q | L | 25 | 5 | U | 7.7 | N | N | N | 3 | Y | 1183 |
| 18 | Q | B | 15 | 11 | U | 11.1 | N | N | N | 3 | Y | 12457.5 |
| 19 | Q | B | 10 | 11 | U | 6.7 | N | N | N | 3 | N | 1764 |
| 20 | Q | B | 21 | 9 | U | 7.7 | N | N | N | 4 | N | 1027.3 |
| 21 | C | Lf | 4 | 4 | U | 0.7 | N | N | N | 2 | N | 19.9 |
| 22 | Q | A | 13 | 13 | U | 3.9 | N | N | Y | 4 | Y | 2272 |
| 23 | Q | C | 4 | 5 | M | 0.5 | Y | N | N | 3 | N | 46.0 |
| 24 | Q | B | 18 | 12 | U | 8.7 | N | N | Y | 3 | Y | 8663.1 |
| 25 | C | Lf | 3 | 5 | U | 0.3 | N | N | N | 1 | N | 4.2 |
| 26 | C | B | 4 | 4 | U | 0.9 | N | N | N | 1 | N | 36.3 |
| 27 | Q | S | 3 | 5 | U | 0.2 | N | N | N | 1 | N | 5.2 |
| 28 | C | B | 18 | 12 | U | 2.3 | N | N | N | 2 | N | 366.9 |
| 29 | Q | B | 5 | 3 | M | 1.5 | N | N | N | 1 | N | 661.8 |
| 30 | C | A | 4 | 22 | M | 1.5 | N | N | N | 4 | N | 179.7 |
| 31 | C | Lf | 16 | 12 | U | 0.2 | N | N | N | 1 | N | 12.7 |
| 32 | C | C | 2 | 4 | M | 0.7 | Y | N | N | 1 | N | 194.8 |
| 33 | Q | Lf | 18 | 10 | U | 1.2 | N | N | N | 3 | N | 3.6 |
| 34 | Q | Lf | 12 | 16 | U | 0.4 | N | N | N | 3 | N | 6.7 |
| 35 | Q | Lf | 2 | 4 | U | 0.7 | N | N | N | 1 | N | 19.9 |
| 36 | Q | Lf | 19 | 9 | U | 1.3 | N | N | N | 3 | N | 26.3 |
| 37 | Q | Lf | 2 | 4 | U | 0.7 | N | N | N | 1 | N | 19.9 |
| 38 | Q | Lf | 20 | 8 | U | 1 | N | N | N | 3 | N | 43.9 |
| 39 | Q | Lf | 20 | 8 | U | 1.3 | N | N | N | 4 | N | 78.9 |
| 40 | Q | Lf | 20 | 8 | U | 1.8 | N | N | N | 2 | N | 1035.6 |
| 41 | Q | Lf | 10 | 14 | U | 0.1 | N | N | N | 1 | N | 1.1 |
| 42 | Q | S | 2 | 5 | U | 0.7 | N | N | N | 1 | N | 81.8 |
| 43 | C | Lf | 16 | 12 | U | 0.3 | Y | N | N | 3 | N | 1.7 |
| 44 | C | S | 11 | 19 | M | 0.9 | N | N | N | 4 | N | 36.3 |
| 45 | C | Lf | 18 | 12 | U | 0.1 | N | N | N | 2 | N | 0.3 |
| 46 | C | A | 4 | 4 | M | 0.8 | N | N | N | 2 | N | 84.9 |
| 47 | C | S | 26 | 4 | U | 6.2 | N | N | N | 4 | N | 73.5 |
| 48 | Q | Lf | 15 | 11 | U | 0.2 | N | N | N | 3 | N | 11.0 |
