# Supplementary material for: Host Niches and Defensive Extended Phenotypes Structure Parasitoid Wasp Communities
Source: PLoS Biol. 2009 Aug 25;7(8):e1000179. doi: 10.1371/journal.pbio.1000179 (PMC2719808; doi:10.1371/journal.pbio.1000179)
Supplement: Table S3 — Parasitoid species list. The full names and family affiliations of all parasitoid species sampled are given below. All are members of the superfamily Chalcidoidea. The families represented are Eulophidae (Eul), Eupelmidae (Eup), Eurytomidae (Eury), Ormyridae (Orm), Pteromalidae (Pter), and Torymidae (Tor). A full list of the parasitoid composition of galls of each host is available from the authors by request. (0.04 MB DOC) [file pbio.1000179.s005.doc]

| **Species** | **No of host gall types** | **Species** | **No of host gall types** |
| --- | --- | --- | --- |
| *Aprostocetus aethiops* (Zetterstedt, 1838) (Eul)  *Aprostocetus biorrhizae* (Szelényi, 1941) (Eul)  *Aprostocetus cerricola* (Erdös, 1954) (Eul)  *Aulogymnus arsames* (Walker, 1838) (Eul)  *Aulogymnus gallarum* (Linnaeus, 1761) (Eul)  *Aulogymnus obscuripes* (Mayr, 1877) (Eul)  *Aulogymnus skianeuros* (Ratzeburg, 1844) (Eul)  *Aulogymnus testaceoviridis* (Erdös, 1961) (Eul)  *Aulogymnus trilineatus* (Mayr, 1877) (Eul)  *Baryscapus anasillus* Graham, 1991 (Eul)  *Baryscapus berhidanus* Erdös, 1954 (Eul)  *Baryscapus diaphantus* (Walker, 1939) (Eul)  *Baryscapus pallidae* Graham, 1991 (Eul)  *Caenacis lauta*. (Walker, 1835)Walker (Pter)  *Cecidostiba fungosa*Geoffroy in Fourcroy, 1785 (Pter)  *Cecidostiba saportai* Graham, 1984 (Pter)  *Cecidostiba semifascia* (Walker, 1835) (Pter)  *Cirrospilus* spp. Westwood, 1832 (Eul)  *Closterocerus trifasciatus* Westwood, 1833 (Eul)  *Eumacepolus obscurior* Graham, 1961 (Pter)  *Eupelmus annulatus* Nees, 1834 (Eup)  *Eupelmus cerris* Förster, 1860 (Eup)  *Eupelmus splendens* Giraud (Eup)  *Eupelmus urozonus* Dalman, 1820 (Eup)  *Eupelmus vesicularis* (Retzius, 1783) (Eup)  *Eurytoma brunniventris* Ratzeburg, 1852 (Eury)  *Eurytoma pistacina* Rondani, 1877 (Eury)  *Hobbya stenonota* (Ratzeburg, 1848) (Pter)  *Megastigmus* sp.1 Dalman, 1820 (Tor)  *Megastigmus dorsalis* (Fabricius, 1798) (Tor) | 17  2  12  7  23  4  15  6  9  4  4  3  18  16  33  1  7  2  1  1  28  1  1  41  7  42  19  12  1  28 | *Megastigmus stigmatizans* (Fabricius, 1798) (Tor)  *Megastigmus synophri* Mayr, 1874 (Tor)  *Mesopolobus amaenus* (Walker, 1834) (Pter)  *Mesopolobus dubius* (Walker, 1834) (Pter)  *Mesopolobus fasciiventris* Westwood, 1833 (Pter)  *Mesopolobus fuscipes* (Walker, 1834) (Pter)  *Mesopolobus sericeus* (Förster, 1770 (Pter)  *Mesopolobus tarsatus* (Nees, 1834) (Pter)  *Mesopolobus tibialis* (Westwood, 1833) (Pter)  *Mesopolobus xanthocerus* (Thomson, 1878) (Pter)  *Minotetrastichus frontalis* (Nees, 1834) (Eul)  *Ormocerus latus* Walker, 1834 (Pter)  *Ormocerus vernalis* Walker, 1834 (Pter)  *Ormyrus nitidulus* (Fabricius, 1804) (Orm)  *Ormyrus pomaceus* (Geoffroy in Fourcroy, 1785) (Orm)  *Pediobius lysis* (Walker, 1839) (Eul)  *Pediobius pyrgo* (Walker, 1839) (Eul)  *Pediobius rotundatus* (Fonscolombe, 1832) (Eul)  *Pediobius saulius* (Walker, 1839) (Eul)  *Sycophila biguttata* (Swederus, 1795) (Eury)  *Sycophila flavicollis* (Walker, 1834) (Eury)  *Sycophila variegata* (Curtis, 1831) (Eury)  *Torymus affinis* (Fonscolombe, 1832) (Tor)  *Torymus auratus* (Müller, 1764) (Tor)  *Torymus cyaneus* Walker, 1847 (Tor)  *Torymus erucarum* (Schrank, 1781) (Tor)  *Torymus flavipes* (Walker, 1833) (Tor)  *Torymus geranii* (Walker, 1833) (Tor) | 6  2  11  2  14  8  3  1  15  16  2  4  6  21  28  3  3  1  1  39  1  13  2  18  13  6  11  6 |
