# Supplementary material for: Host Niches and Defensive Extended Phenotypes Structure Parasitoid Wasp Communities
Source: PLoS Biol. 2009 Aug 25;7(8):e1000179. doi: 10.1371/journal.pbio.1000179 (PMC2719808; doi:10.1371/journal.pbio.1000179)
Supplement: Table S4 — Gall scores for response (community) and sampling variables for pooled-sites analyses in each of (i) sexual generation galls, and (ii) asexual generation galls. Key to variables: Galls producing, total number of galls producing parasitoids; No. emerged, total number of emerging parasitoids; Richness, species richness; MDS axes 1–3 are values for three mutually independent MDS axes describing community composition. (0.11 MB DOC) [file pbio.1000179.s006.doc]

| Parasitoid community property: Sexual generation | | | | | | |
| --- | --- | --- | --- | --- | --- | --- |
| Gall type | Richness | MDS axis 1 | MDS axis 2 | MDS axis 3 | No. emerged | Galls producing |
| 2 | 4 | -0.43 | -1.33 | 0.3 | 85 | 5 |
| 7 | 20 | -0.4 | -0.15 | 0.46 | 115 | 57 |
| 10 | 17 | -1.68 | 0 | 0.24 | 432 | 87 |
| 11 | 14 | -0.76 | 0.11 | -0.46 | 149 | 133 |
| 16 | 17 | 0.09 | -0.1 | 0.33 | 225 | 195 |
| 21 | 17 | -1.23 | -0.03 | 0.78 | 320 | 136 |
| 23 | 15 | -0.91 | -1.03 | -0.02 | 1934 | 203 |
| 25 | 18 | -1.19 | 0.63 | 0.31 | 63 | 58 |
| 26 | 14 | -1.08 | 0.25 | 0.26 | 95 | 52 |
| 27 | 10 | 0.44 | 0.48 | 0.77 | 51 | 40 |
| 29 | 30 | -0.61 | -0.7 | -0.56 | 12507 | 874 |
| 32 | 23 | -1.16 | -0.38 | -0.12 | 1296 | 245 |
| 35 | 5 | -1.75 | 0.04 | -0.91 | 33 | 32 |
| 37 | 3 | -1.4 | 0.89 | -0.24 | 6 | 3 |
| 42 | 18 | -1 | 0.99 | -0.29 | 192 | 153 |
| 46 | 22 | -0.32 | -0.49 | -0.01 | 630 | 339 |
| 47 | 8 | 0.05 | 0.34 | 1.26 | 46 | 42 |

| **(b) Parasitoid community property**: **Asexual generation** | | | | | | |
| --- | --- | --- | --- | --- | --- | --- |
| Gall type | Richness | MDS axis 1 | MDS axis 2 | MDS axis 3 | No. emerged | Galls producing |
| 1 | 16 | 0.43 | -0.28 | 0.29 | 665 | 271 |
| 3 | 8 | 0.87 | 0.49 | 0.24 | 48 | 30 |
| 4 | 23 | 0.06 | -0.2 | -0.42 | 486 | 182 |
| 5 | 13 | 0.68 | 0.26 | -0.16 | 129 | 90 |
| 6 | 7 | 0.82 | 0.67 | -0.26 | 52 | 41 |
| 8 | 19 | 0.22 | -0.36 | 0.21 | 2336 | 509 |
| 9 | 9 | 0.66 | -0.1 | -0.04 | 129 | 78 |
| 12 | 13 | 0.44 | -0.11 | -0.05 | 178 | 86 |
| 13 | 3 | 0.83 | -0.46 | 0.08 | 7 | 7 |
| 14 | 13 | 0.34 | -0.33 | 0.05 | 111 | 87 |
| 15 | 21 | 0.36 | -0.58 | 0.47 | 1293 | 333 |
| 17 | 8 | 0.36 | 0.81 | 0.18 | 37 | 18 |
| 18 | 13 | 0.16 | 0.13 | -0.32 | 606 | 201 |
| 19 | 22 | 0.76 | 0.39 | 0.12 | 478 | 166 |
| 20 | 12 | 0.41 | 0.22 | 0.15 | 251 | 156 |
| 22 | 19 | -0.04 | -0.23 | -0.96 | 415 | 170 |
| 24 | 8 | 0.34 | -1.16 | 0.08 | 55 | 53 |
| 28 | 14 | 0.11 | 0.47 | 0.5 | 156 | 53 |
| 30 | 10 | 0.01 | -0.53 | 0.76 | 63 | 42 |
| 31 | 12 | 0.65 | 0.59 | -0.52 | 92 | 85 |
| 33 | 9 | 0.57 | 0.25 | -0.27 | 58 | 55 |
| 34 | 13 | 0.09 | 0.57 | -0.17 | 44 | 43 |
| 36 | 7 | 0.63 | -0.5 | -0.24 | 40 | 39 |
| 38 | 12 | 0.39 | -0.77 | -0.4 | 61 | 60 |
| 39 | 18 | 0.77 | -0.3 | -0.78 | 431 | 421 |
| 40 | 20 | 0.53 | -0.1 | -0.67 | 363 | 332 |
| 41 | 12 | 0.51 | 0.59 | -0.37 | 77 | 74 |
| 43 | 12 | -0.07 | 0.59 | 0.09 | 39 | 39 |
| 44 | 14 | 0.46 | 0.01 | 0.65 | 187 | 50 |
| 45 | 14 | 0.22 | 0.4 | -0.36 | 116 | 95 |
| 48 | 8 | 0.75 | 0.07 | 0.04 | 32 | 29 |
